# Inflammatory fibroid polyp (Vanek's tumor) causing double compound ileo-ileal intussusception in an adult patient, a case report

**DOI:** 10.1016/j.ijscr.2022.106947

**Published:** 2022-03-16

**Authors:** Ahmed Gadoura, Farah Mohammed, Mohamed Abdulkarim, Ammar Ibn Yasir, Dafalla Shani, Nadir Salih

**Affiliations:** a Alzaiem Alazhari University, Department of Surgery, Sudan; b National Ribat University, Sudan

**Keywords:** Sudan, case report, Fibroid polyp, Ileo-ileal, Adult, Double-intussusception

## Abstract

**Introduction and importance:**

The majority of the of small bowel intussusception causes are benign, and different types of benign tumors have been reported as the lead points of an intussusception. Among these, some studies have reported inflammatory fibroid polyp as a cause of small bowel intussusception. However, according to our knowledge this is the first case to be reported in Africa of an adult double compound intussusception due to an inflammatory fibroid polyp.

**Case report:**

A 32-year-old female presented with epigastric abdominal pain, nausea, vomiting and diarrhea for 2 weeks. Investigations revealed a small bowel obstruction due to intussusception, and exploratory laparotomy was planned.

**Clinical discussion:**

Inflammatory fibroid polyp causing this unique feature of double compound ileo-ileal intussusception should be observed in adult patients who presents with intestinal obstruction. A CT scan is the diagnostic modality of choice, and we think that the disease is the first of its kind to be reported in Africa.

**Conclusions:**

Double ileo-ileal intussusception is a rare cause of intestinal obstruction in adult patients.

**Interventions and outcome:**

Laparotomy reviled an ileo-ileal intussusception. After manual reduction of this intussusception, another intussusception was observed and containing a polyp. A clear margin resection and end-to-end primary anastomosis were performed. The histopathological report established the diagnosis of Inflammatory Fibroid Polyp. After the procedure, the patient's condition improved well with no complications.

**Methods:**

This case report has been reported in line with the SCARE Criteria (Agha et al., 2020 [1]).

## Highlights

• Intussusception is a condition in which there is telescoping ladder f one bowel segment into another resulting in mechanical bowel obstruction.
• While intussusception is considered a common condition in infants and young children, it is rare in adults. It is not common to have two lead points (Double intussusception) for the disease, causing symptoms simultaneously.
• In this case, we report a case of double intussusception in an adult patient, something that has been reported in only handful of cases worldwide.

## 1 Introduction

Small bowel intussusception is a rare condition that is commonly caused by benign tumors, including lipomas, leiomyomas, neurofibromas and adenomas [2]. Intussusceptions are caused by several etiologies, and these etiologies vary according to the age of the patient and the site of the intussusception. In pediatric patients 90% of cases are idiopathic, while in adults 70–90% of the cases are due to organic causes (tumors or adhesive disease) [3], [4].

## 2 Rationale

Inflammatory fibroid polyp is a rare gastrointestinal benign tumor, and several studies have reported it as a cause of small bowel intussusception [5], [6], [7], [8]. However, according to our knowledge this is the first reported case of small bowel double compound intussusception in adults to be caused by this tumor.

## 3 Patient information

A 32-year-old female presented to the accident and emergency department complaining of epigastric abdominal pain, nausea, vomiting and diarrhea for 2 weeks with no history of similar condition, hospitalization or chronic illness. There is no family history of similar condition.

## 4 Clinical findings

On examination, the patient vital signs were normal apart from tachycardia. Regarding her abdominal examination, there was slight abdominal distention and epigastric tenderness. Her cardiovascular and respiratory systems were normal.

## 5 Timeline

Initially, our patient presented to the emergency department complaining of abdominal pain, nausea, and vomiting. After stabilizing the patient by the ER team, she was admitted to the medical ward and treated conservatively for two days. After failure of the conservative management, we have been called in the surgical department for consultation. Laboratory and imaging investigations confirmed her diagnosis of small bowel obstruction, and surgical intervention was decided.

## 6 Diagnostic assessment and interpretation

**Fig. 1:**
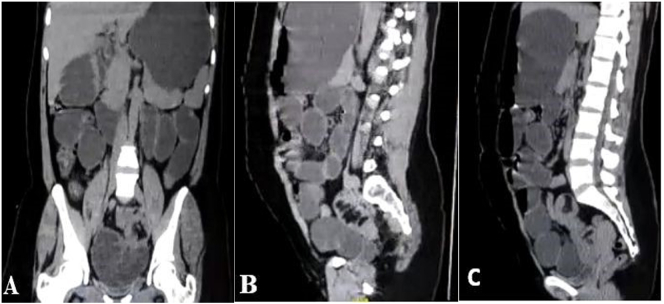
(A) Coronal section CT scan showing grossly dilated stomach and small bowel loops. (B) and (C) Sagittal sections showing bowel within bowel configuration, evident by presence of small bowel mesentery within bowel lumen.

Initial laboratory investigations showed elevated blood urea, high serum creatinine levels, and leukocytosis. Abdominal radiograph was requested and revealed a small bowel dilatation. An abdominal ultrasound was performed, showing multiple dilated small bowel loops with air fluid level. A plain abdominal CT was performed which revealed a grossly dilated stomach and small bowel loops with collapsed large bowel (Fig. 1: A). A transitional point at the distal third of the terminal ileum was seen in the central lower abdominal region, just above the urinary bladder, where there was a bowel-within-bowel configuration, evident by presence of small bowel mesentery within the bowel lumen. This appearance is consistent with small bowel obstruction due to ileo-ileal intussusception (Fig. 1: B, C).

## 7 Intervention

A diagnosis of gastropathy due to acute kidney injury and sepsis was made, and the patient was admitted to the medical ward and managed with intravenous fluids, proton pump inhibitors, and antibiotics. Two days after failure of conservative management and imaging results revealed, a laparotomy was guaranteed.

**Fig. 2:**
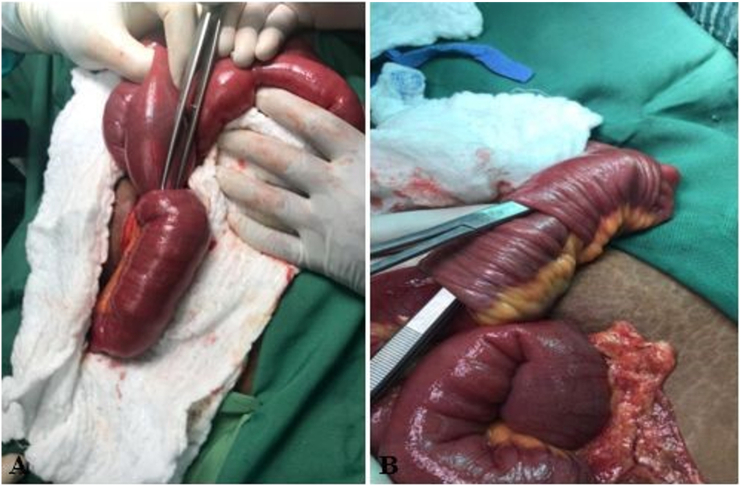
Well defined polypoid lesion.

Intraoperatively, intussusception was identified within 25 cm from the ileocecal valve (Fig. 2.A). After manual reduction of this intussusception, another intussusception was observed (Fig. 2.B). There were multiple enlarged lymph nodes along the small bowel mesentery, a feature suggestive of tumor or lymphoma. An 80 cm resection with clear margins was done in about 15 cm proximal to the ileocecal valve, and an end-to-end primary anastomosis was performed. The resected segment and lymph-nodes were sent for histopathological assessment.

Operator: A senior consultant of general surgery. He attained his fellowship at the Royal College of Surgeons in Ireland in 2002. The operation was done in a private hospital in Khartoum state.

## 8 Follow-up and outcome

After the operation, the patient's condition improved with no complications, and she was discharged on day 7. She came for a follow-up visit one week after her discharge with no complications apart from mild diarrhea. Follow up after 2 weeks revealed no complications and the wound was healing well. The resected segment was sent for histopathological analysis which revealed the following: a dilated area in the bowel measured 6 × 6 × 3 cm, surface cut showed a 4 × 3 cm well-defined polypoid lesion, a mesenteric root measuring 7 × 1 cm (Fig. 2).

**Fig. 3:**
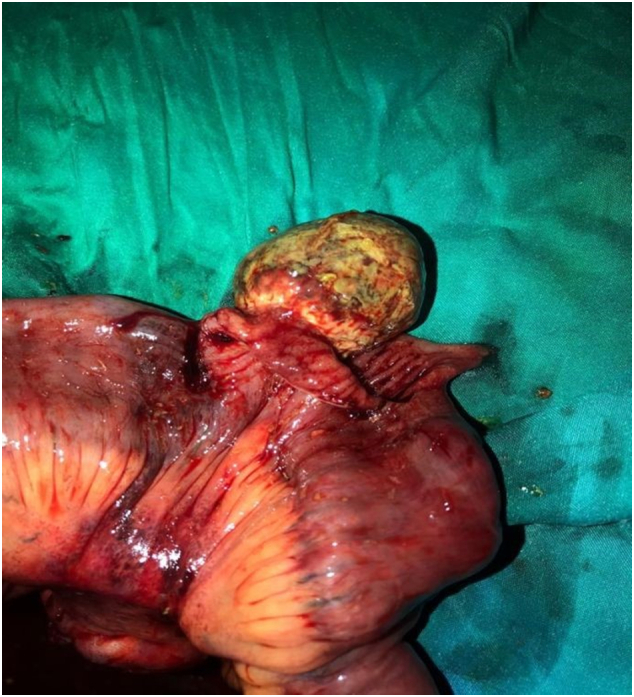
(A) Showing the ileo-ileal intussusception before manual reduction. (B) Showing the second intussusception after manual reduction.

Microscopic examination sections showed that the lesion was composed of loosely homogenous and vascular stroma. There was a perivascular condensation of myofibroblasts. Background stroma showed a mixture of inflammatory cells composed mainly of lymphocytes and plasma cells. In addition, foci of hemorrhage and congestion were noted. Additionally, the mucosa and lamina propria displayed mild inflammation. Furthermore, no apparent evidence of dysplasia or malignancy, and the mesenteric lymph nodes showed reactive follicular hyperplasia. All of these features were consistent with the diagnosis of inflammatory fibroid polyp (Vanek's tumor) (Fig. 3: A and B).

## 9 Discussion

Inflammatory fibroid polyp is a benign gastrointestinal tumor, that was described originally as “gastric submucosal granuloma with eosinophilic infiltration” in 1949 by Vanek [9]. It commonly occurs among older adults in the fifth decade and has a slight predominance among females. This tumor usually affects the stomach followed by the small bowel and rarely the large bowel. Patients could present with gastrointestinal bleeding, abdominal pain and other symptoms of obstruction, and could present emergently as intussusception. Sometimes it could be discovered incidentally in asymptomatic patients.

It appears commonly as an intraluminal pedunculated polypoid lesion. Inflammatory fibroid polyp sizes ranges from few millimeters up to more than 10 cm, it is often a submucosal tumor with ill-defined margins and fleshy appearance.

Histologically, they are composed of stellate, short spindled, or epithelioid cells, that are arranged randomly in a myxoid or edematous stroma, with an inflammatory infiltrate which consists mainly of eosinophils, as well as prominent small blood vessels and capillaries. Moreover, most of these tumors are positive for CD34 [10], [11].

Intussusception accounts for 0.003% to 0.02% of all hospital admissions, which makes it a rare condition [12]. Despite the fact that Intussusception can present with many nonspecific symptoms and can have an acute or chronic course, the clinical presentation of intussusception is usually with abdominal pain, palpable abdominal mass (in 50% of the patients), nausea and vomiting [13]. Intussusceptions are generally classified according to the location of the lead point into a colonic group which includes: appendicico-cecal, sigmoido-rectal, colo-colonic and ileocecal-colic intussusceptions, or an enteric group. The enteric group includes: ileocolic, ileoileal (this case), and jejuno-jejunal intussusceptions [14]. Double intussusception is a rare form of intussusception, and it has four types: double site intussusception, double compound intussusception (this case), which is extremely rare, separate intestines telescoping into the same distal intestine, and double telescoping of the distal and proximal intestine through a patent duct [15]. Several modalities can be used to diagnose intussusception; however, computed tomography scan is the best diagnostic modality, followed by ultrasound, and both of them show the characteristic features of intussusception which is sausage or target shape mass [16].

Regarding the surgical management of intussusception, it still debatable between the options of reduction of intussusception lesion or perform an “en-bloc” resection without reduction. This debate originates from the fact that there is a possibility of malignant tumor being the cause of intussusception, especially with the colonic intussusceptions. Some advocated reduction before resection. However, most surgeons agree that resection is mandatory especially with older patients and colonic intussusceptions, a third group of surgeons recommended a selective approach depending on the location of the intussusception and the degree of malignancy suspicion [17]. Each method has advantages and disadvantages; resection without reduction decreases the risk of tumor dissemination and spread, and reduction before resection decreases the risk for short-bowel syndrome [16].

## Patient perspective

Patient stated that she is now back to her normal healthy life, without any postoperative complications.

## Provenance and peer review

Not commissioned, externally peer-reviewed.

## Sources of funding

Authors received no funding from any individual or institution and this work is completely a voluntary work.

## Ethical approval

Ethical approval was obtained from Ethical committee at Alzaiem Alazhari University.

## Consent

Written informed consent was obtained from the patient for publication of this case report and accompanying images. A copy of the written consent is available for review by the Editor-in-Chief of this journal on request.

## Author contribution

1. Ahmed Mohamed Ali: Involved in study design, data acquisition, drafting the article, revising it critically and finally approved the manuscript.
2. Farah Mohammed: Involved in study design, data acquisition, drafting the article, revising it critically and finally approved the manuscript.
3. Ammar Ibn Yasir: Involved in conception of the study design, drafting the article and finally approved the manuscript.
4. Mohamed Abdulkarim: Involved in conception of the study design, drafting the article and finally approved the manuscript.
5. Dafalla Shani: Involved in the design of the study, revising it critically and finally approved the manuscript.
6. Nadir Salih: Involved in the design of the study, revising it critically and finally approved the manuscript.

## Research registration

Not applicable.

## Guarantor

Ahmed Mohamed Ali.

## Declaration of competing interest

Authors report no conflict of interest of any sort.
